# The Dissemination of Mass in the United States: Results and Implications of Recent BIPM Calibrations of US National Prototype Kilograms

**DOI:** 10.6028/jres.119.001

**Published:** 2014-03-12

**Authors:** Zeina J Kubarych, Patrick J Abbott

**Affiliations:** National Institute of Standards and Technology, Gaithersburg, MD 20899

**Keywords:** BIPM calibration, calibration, dissemination, kilogram, mass metrology, mass stability, national prototypes, national standards, platinum-iridium kilograms, recalibration of US national standard, SI unit of mass

## Abstract

The National Institute of Standards and Technology (NIST) is responsible for the dissemination of the unit of mass within the United States of America through the national prototype kilogram K20 and its check standard K4. These platinum-iridium artifacts have been in use since 1889 and are periodically sent to the International Bureau of Weights and Measures (BIPM) for recalibration. The following is a brief description of the roles of the national prototype kilograms in the dissemination of mass in the United States of America, and the implications for NIST mass calibration customers of the most recent recalibrations of K20 and K4.

## 1. Introduction

The National Institute of Standards and Technology (NIST) is responsible for the dissemination of the unit of mass, the kilogram, in the United States of America. The kilogram is one of seven fundamental units of measure comprising the International System of Units (SI), and is defined as follows [1]:

The kilogram is the unit of mass; it is equal to the mass of the international prototype of the kilogram.

The international prototype of the kilogram (IPK) is kept in a vault at the International Bureau of Weights and Measures (BIPM) located in Sèvres, just outside of Paris, France, and is taken out only rarely for comparison with its official copies. The definition of the kilogram implies that the IPK always has a mass of exactly one kilogram with no uncertainty; however, due to the inevitable accumulation of contaminants on surfaces, the international prototype is subject to reversible surface contamination that approaches 1 µg per year in mass [2]. For this reason, the CIPM (International Committee of Weights and Measures) declared that, pending further research, the reference mass of the international prototype is that immediately after cleaning and washing by a specified method [3–4]. This reference mass is used to calibrate national standards made of platinum-iridium alloy [2].

The United States of America’s national prototype kilogram is designated as K20 and its check standard mass is K4, both made of platinum-iridium alloy (Pt-90 %, Ir-10 %). Two other prototypes, K79 and K92, were purchased in 1996 and 2008, respectively, and serve as additional checks for K20. More information on the history of the IPK and the K20 and K4 prototypes is given by Jabbour and Yaniv [5]. NIST is responsible for the storage and maintenance of the national prototype kilogram and its check standards, and uses these artifacts to calibrate its own working standards for the dissemination of mass from 1 mg to 28 000 kg. Proper maintenance of the standards includes periodic recalibration at the BIPM. There is no prescribed recalibration interval for the national standard K20, and during its more than 120 year existence, periods of several decades have elapsed between recalibrations. Table 1 shows the dates of recalibration for all four Pt-Ir kilograms belonging to NIST, along with their corrections from the IPK.

## 2. Dissemination of the Unit of Mass

Since the unit of mass is only available at the BIPM, it must be disseminated to the rest of the world through a series of comparison calibrations involving national prototypes. For the United States, K20 is calibrated at the BIPM, and the unit of mass is then disseminated at NIST from K20 to a mass scale covering multiples and submultiples of the kilogram, as outlined in Ref. [5]. Stainless-steel 1 kg working standards are first calibrated for use by direct comparison with K20; these working standards are then used to calibrate stainless-steel multiples and submultiples of the kilogram. Transferring the mass scale to stainless-steel working standards minimizes the use and handling of the platinum prototypes, thereby protecting them from possible damage or contamination (dirt, grease, etc.) that could lead to instability.

Prior to any dissemination, the mass of K20 is verified at NIST through the use of check standard K4, and more recently, K79 and K92. In this process, the check standards are calibrated against K20, and the results are compared against historical values from the BIPM and previous internal verifications [6]. Statistical process controls are applied to the check standard calibration data for quality assurance [7]. Deviations from accepted mass values of the check standards may indicate a change in the national prototype K20 or in one or more of the check standards. Redundancy of measurements is essential in identifying possible instabilities in any given mass artifact, and control charts of results are used to determine when recalibration at the BIPM is necessary.

## 3. Calibration History of Platinum Prototypes

**Fig. 1:**
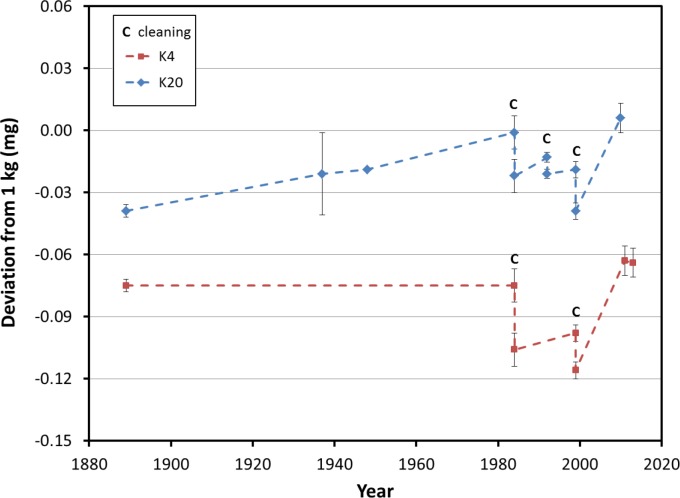
History of the calibration of the U.S. National Prototype K20 and check standard K4 at the BIPM. The uncertainty bars (k=1) for each number are the uncertainties reported by the BIPM on the calibration certificates. No uncertainty was reported on the 1948 calibration of K20.

Plots of the BIPM calibration histories for K20 and K4 are shown in Fig. 1. The plots indicate several salient features pertaining to stability of the artifacts. The calibration history of K20 indicates that between 1889 and 1984 there was very little change in its mass, approximately 0.020 mg. After 1983, calibrations were performed more frequently, and cleaning of the artifact (marked by a **C** in the plots) was done on three occasions. The same is true for K4, which was recalibrated in 1984 for the first time in nearly a century.

### 3.1 Recent Recalibrations

**Fig. 2:**
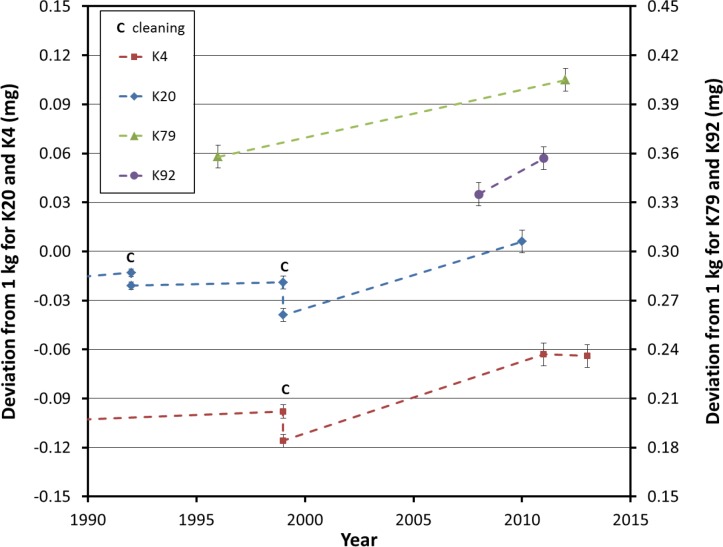
History of the calibration of all four Pt-Ir prototypes at the BIPM since 1990. The K4 and K20 data is identical to the last three calibrations shown in Fig. 1. The uncertainty bars (k=1) for each number are the uncertainties reported by the BIPM on the calibration certificates.

In 2010, K20 was sent to the BIPM for recalibration after approximately 10 years since its previous calibration. This was soon followed by K4 in early 2011. No cleaning was done on either artifact. As seen in Fig. 1, both artifacts changed significantly in magnitude (+0.045 mg for K20 and +0.053 mg for K4) and in the same direction (heavier). These changes are unprecedented in the history of both artifacts over such a short recalibration interval, though ironically, these changes were not detected at NIST. This is due to the comparison nature of mass metrology and the nearly identical change in magnitude and direction of the artifacts’ masses. The prototypes K79 and K92 were also recalibrated at the BIPM in 2012 and 2011, respectively. K92 was found to be 0.022 mg heavier than its initial value reported in 2008, and K79 was found to be 0.047 mg heavier than its most recent weighing in 1996. The changes to these prototypes are also very large when compared to the historical data of K20 and K4 presented in Fig. 1. Prototype kilogram K4 was recalibrated in 2013 and it was found to be 0.001 mg lighter than the 2011 value. The calibration history for all four Pt-Ir prototypes, limited to the period of time from 1990 to 2013, is shown in Fig. 2.

As stated above, K20 and K4 changed by nearly the same amount between 1999 and 2011. During that time period, K79 changed by a similar amount. The average rate of change for the artifacts K20, K4, and K79 over this time period is 0.0041 mg/year, 0.0044 mg/year, and 0.0029 mg/year, respectively. Although it is not valid to assume that the changes in the prototypes occurred smoothly and monotonically between 1999 and 2011, the fact that K4 and K79 didn’t vary by more than ±0.004 mg when used as check standards for K20 during this period is further evidence of a similar rate of change for all three prototypes. Of course, the lack of calibration data points in the middle of the 12 year interval from 1999 to 2011makes it impossible to draw other conclusions about the rate of mass change of the prototypes. The rate of change of 0.0073 mg/year exhibited by K92 during a three year period from 2008 to 2011 is unexpectedly large and therefore very alarming.

### 3.2 Effect of Recalibration on Mass Dissemination and Recommendations

The large changes in K20 and K4 are reflected in all subsequent calibrations performed by NIST. The following are implications and recommendations that pertain to NIST’s mass calibration customers:

1. As a result of the recalibration of the US prototype kilogram all calibrations performed at NIST after December 2010 could show a +0.045 mg/kg change in a standard being calibrated, in addition to other changes due to wear, contamination, and any other effects contributing to the stability of mass standards.
2. Since it is not possible to determine the origin in time or the nature of the +0.045 mg/kg change one cannot retrospectively correct any mass standard calibrated prior to December 2010.
3. In consequence, laboratories operating at Echelon I [8] (OIML [9] classes E_1_, E_2_, ASTM [10] classes 0, 1) should have their references recalibrated at NIST to guarantee traceability to the 2010 calibration of K20.
4. Laboratories operating at Echelon I should have their check standards recalibrated at NIST or alternatively have them recalibrated internally with respect to their post December 2010 recalibrated references (step 3). Failure to recalibrate the check standards may result in failed statistical process control tests as the newly obtained values of the check standards are compared to values based on a different traceability chain (1999 calibration of K20).
5. As recalibrations can take many months to implement, accreditation bodies must take this information into account during the accreditation assessment process (e.g., evaluation of proficiency testing results and traceability claims).
6. Laboratories operating at Echelon II [8] (OIML classes F_1_, F_2_ and ASTM classes 2, 3) or Echelon III [8] (OIML classes M_1_, M_2_, M_3_, ASTM classes 4, 5, 6, 7, NIST class F [11]) should be largely unaffected by this change.

## 4. Conclusion

Given the unprecedented change in mass, NIST will calibrate K20 on a more regular basis to maintain tighter control on the traceability to the international prototype kilogram. Within the past two years, K20, K4, K79, and K92 were sent to BIPM for calibration. K4 was calibrated at the BIPM again in 2013, and its mass is well within the uncertainty of the comparison measurements with K20 performed at NIST.

## Figures and Tables

**Table 1:** Year of calibration and masses reported by BIPM for the U.S. prototypes showing corrections after cleaning (when cleaned) in mg to that of the international prototype kilogram (IPK).

Year	K20	K4	K79	K92
1889	−0.039	−0.075		
1937	−0.021			
1948	−0.019			
1984	−0.022	−0.106		
1992	−0.021			
1996		+0.358		
1999	−0.039	−0.116		
2008				+0.335
2010	+0.006			
2011		−0.063		+0.357
2012			+0.405	
2013		−0.064		
